# Experience with abatacept in refractory lupus nephritis

**DOI:** 10.1007/s00296-023-05389-0

**Published:** 2023-08-31

**Authors:** Emma Calatayud, Marco Montomoli, Ana Ávila, Asunción Sancho Calabuig, Juan José Alegre-Sancho

**Affiliations:** 1 Hospital Universitario Doctor Peset, Valencia, Spain; 2 Fundación para el Fomento de la Investigación Sanitaria y Biomédica de la Comunidad Valenciana (FISABIO), Valencia, Spain; 3 Hospital Clínico Universitario de Valencia, Valencia, Spain; 4 Department of Medicine, University of Valencia, Valencia, Spain

**Keywords:** Lupus nephritis, Abatacept, CTLA-4-Ig, Biologic therapy

## Abstract

Lupus nephritis is a major cause of morbidity in patients with systemic lupus erythematosus. Responsiveness to treatment is crucial to avoid chronic kidney disease. New molecules have been developed in recent years to improve renal survival rates. Biological therapies as coadjutant to conventional induction treatment have been tested in randomized clinical trials with heterogeneous results. Like many others biologic therapies, Abatacept has not shown a clear benefit in the context of clinical trials. We present two cases of lupus nephritis patients in whom addition of abatacept resulted in complete remission of the renal disease. The first case described a 49-year-old male with class IV lupus nephritis with nephrotic range proteinuria and high immunological activity refractory to conventional treatment with cyclophosphamide and corticosteroids and multitarget therapy with tacrolimus, mycophenolate mofetil and prednisone. Several biological therapies (rituximab, belimumab and tocilizumab) were unsuccessfully tried, so that abatacept was added to his background multitarget therapy showing complete clinical response. The second case described a 52-year-old female with class IV lupus nephritis treated initially with conventional treatment with partial response. In successive renal flares with nephrotic proteinuria, she showed intolerance to rituximab and refractoriness to voclosporin. Finally, abatacept was added to her background therapy with MMF and PDN showing complete and maintained remission of the disease. In no case the use of abatacept was associated with serious adverse events. Based on our experience, abatacept should be considered as a safe rescue therapy in patients with refractory lupus nephritis and proteinuria with nephrotic range. In addition to this case, we reviewed the use of abatacept in lupus nephritis in the literature.

## Introduction

Lupus Nephritis (LN) develops in ~ 40% of patients with systemic lupus erythematosus (SLE) [1]. Furthermore, 10–30% of patients with LN progress to end-stage renal disease (ESRD) becoming candidates for renal replacement therapy (RRT) [1, 2]. Achieving a complete clinical response (CR) is crucial to ensure long-term renal survival. Patients who achieve CR have a renal survival of 92% at 10 years, while this is 43% in those with partial response (PR) and only 13% in refractory disease [3].

Currently, the treatment of LN still shows suboptimal results. The renal survival rate has remained constant in the past two decades, suggesting the need to find new therapeutic strategies for those patients who are refractory to conventional treatment. Advances in the knowledge of the different immunopathogenic pathways involved in LN have allowed the development of multiple clinical trials in recent years, which have helped to obtain the approval of different drugs. Abatacept (ABT) is a biologic therapy approved to treat different inflammatory arthropathies [4]. ABT inhibits the activation of T lymphocytes through the blockade of the CD28-CD80/86 stimulation pathway. The utility of ABT in the field of LN has been tested in different studies without a clear benefit of its use despite the improvement observed in different parameters analyzed, such as proteinuria and immunological parameters [5]. We present two cases of refractory LN, where the addition of ABT allowed to achieve total remission of the disease.

### Case 1

A 49-year-old male under joint follow-up by Nephrology and Rheumatology consultation for SLE with WHO Class IV LN diagnosed by kidney biopsy in February 2005. The illness debuted with myocardial involvement, proteinuria (2.2 g/day measured by protein in urine-24 h excretion), microscopic hematuria and acute renal failure with serum creatinine (sCr) of 1.4 mg/dL. He showed serum antinuclear (ANA) positivity (1:1240), serum anti-dsDNA-, anti-Ro > 245 IU/mL and anti-RNP > 32 IU/mL. Initially, he received 3 pulses of 500 mg of 6-methylprednisolone (6-MP), 9 pulses (6 monthly pulses and 3 quarterly pulses) of intravenous (IV) cyclophosphamide (CYC) (1 g/m^2^), with double blockade of the renin-angiotensin system (RAS). Seven months after this treatment, he achieved a PR (proteinuria 0.68 g/day). However, during quarterly IV CYC pulses, the patient presented a relapse with nephrotic-range proteinuria (4.5 g/day), which was treated with 3 additional pulses of 6-MP followed by oral prednisone (PDN) (1 mg/kg) in tapering regimen and mycophenolate mofetil (MMF) 2 g/day, achieving a transient PR (0.7 g/g) in September 2006.

One year later (August 2007), due to a new renal flare (7.7 g/day), “off label” rituximab (RTX) was added to the background treatment (MMF 2 g/day and PDN 5 mg/day) in 4 weekly infusions at doses of 375 mg/m^2^. From 2007 to 2011 he received 4 more cycles of RTX (1000 mg every 14 days) achieving a transient PR (0.7 g/g) in July 2009.

Due to refractoriness to RTX, tacrolimus (TAC) was added during this time (January 2010). Six months after the start of multitarget therapy (RTX, TAC, MMF and PDN), proteinuria was stabilized at around 2–3 g/day. Nevertheless, in September 2011, proteinuria gradually increased again to nephrotic range and anti-dsDNA turned positive. Due to its persistence, it was decided to change RTX to Belimumab (BEL), as an off-label indication.

After 6 months of treatment with BEL (8 monthly infusions of 10 mg/kg) and multitarget therapy, nephrotic-range proteinuria (10 g/day) and immunological activity were still present. Due to the lack of responsiveness, it was decided to perform a new kidney biopsy. Anatomopathological findings showed persistence of LN class IV with an activity index of 10/24 and a chronicity index of 2/12, with glomerulosclerosis < 10%. Electron microscopy revealed diffuse podocyte fusion, which was related to the degree of proteinuria.

In the absence of other therapeutic alternatives, it was decided to abandon BEL and associate subcutaneous Tocilizumab (TCZ) (162 mg/week) to the multitarget therapy, as an off-label indication. The response was disappointing; the patient presented renal function deterioration for the first time (sCr 1.42 mg/dL) with persistent nephrotic-range proteinuria (12 g/day), active sediment (100–150 RBC/µL), hypoalbuminemia (2.5 g/dL) and complement consumption.

**Fig. 1:**
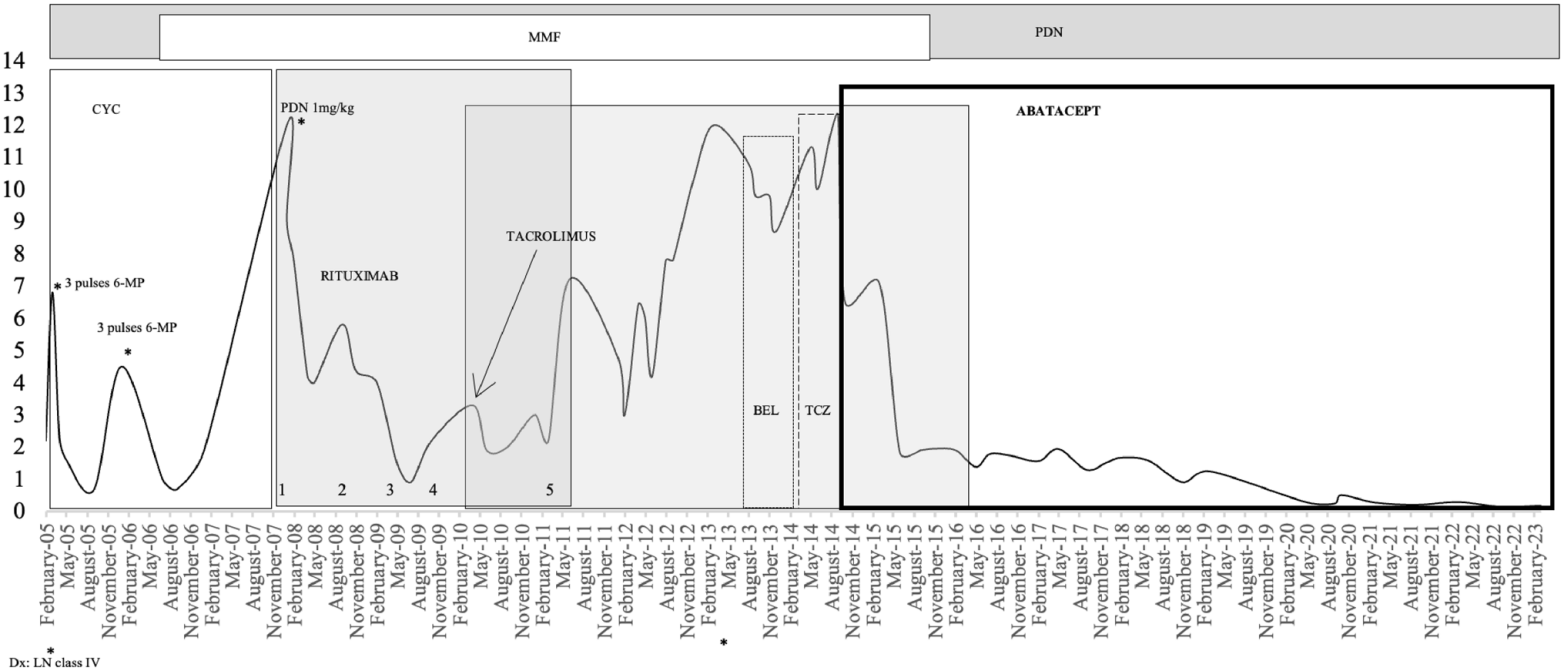
Proteinuria evolution measured by 24 h-urine excretion (g/day)

Given the refractoriness of the LN, in September 2014, off-label subcutaneous ABT (125 mg weekly) was added to his maintenance treatment (TAC, MMF, and PDN). Three months later, proteinuria decreased by half (6.5 g/day) with sediment normalization and renal function recovery (sCr 1.18 mg/day). Over the years proteinuria progressively decreased until achieving CR 6 years after ABT initiation: proteinuria 0.5 g/day, inactive sediment (10–20 RBC/µL), normal renal function and complement levels normalization and decrease of immunology activity (Fig. 1). Both TAC and MMF were gradually reduced until their complete withdrawal, while he has maintained treatment with low-dose oral prednisone (5 mg). The patient has remained clinically asymptomatic until this moment under ABT treatment with no infectious complications or hospital admissions derived from the disease and/or treatment.

### Case 2

A 52-year-old female with SLE diagnosed at the age of 26 (1996). She debuted with polyarthritis, oral aphthae, alopecia, malar erythema, and Raynaud’s phenomenon. At the beginning of her illness, she also presented nephrotic-range proteinuria, so a kidney biopsy was performed, which showed WHO class IV LN. The patient was treated at another center with IV CYC and 6-MP pulses followed by oral PDN. Azathioprine and chloroquine were not tolerated. So, MMF (1 g/day) was added to oral PDN and maintained from 2007 to 2010. One year later (2011), nephrotic-range proteinuria was detected, so off-label RTX was started. However, the treatment was withdrawn when she presented a serum sickness syndrome after the second RTX dose.

In 2012, she started follow-up in our hospital in the interdisciplinary consultation for Rheumatology and Nephrology. At that time, she was still receiving treatment with PDN 10 mg/day, and she had abandoned MMF for no clear reason. Laboratory findings showed normal renal function (sCr 0.75 mg/dL), proteinuria (1.5 g/g), consumption of complement (C3: 60 mg/dL and C4: 8 mg/dL) and positive anti-dsDNA (14 IU/mL). In 2016, due to a renal flare (proteinuria 4 g/day), MMF (2 g/day) was reintroduced, achieving PR (2 g/day).

In April 2018, after performing an update biopsy that confirmed WHO LN class IV (activity index 16/24, chronicity index 6/12), the patient was included in the phase III study, AURORA (AURinia Orelvo Renal Assessment, “A randomized, controlled, double-blind clinical trial comparing the efficacy and safety of Orelvo (Voclosporin 24.6 mg/12 h) versus placebo”. Throughout the trial, MMF, PDN (minimum dose 2.5 mg), and RAS blockers were continued.

In January 2020, the patient was withdrawn from the trial due to nephrotic syndrome (hypertension, edema, and proteinuria (UPCRs 3.9 g/g). It was decided to resume IV CYC in the Eurolupus-2 regimen (600 mg/14 days × 6 doses) and the PDN was increased to a dose of 1 mg/kg/day (50 mg/day). After the first CYC infusion, the patient referred gastrointestinal intolerance and she expressed her wish to stop the treatment, so she only received one single CYC dose.

**Fig. 2:**
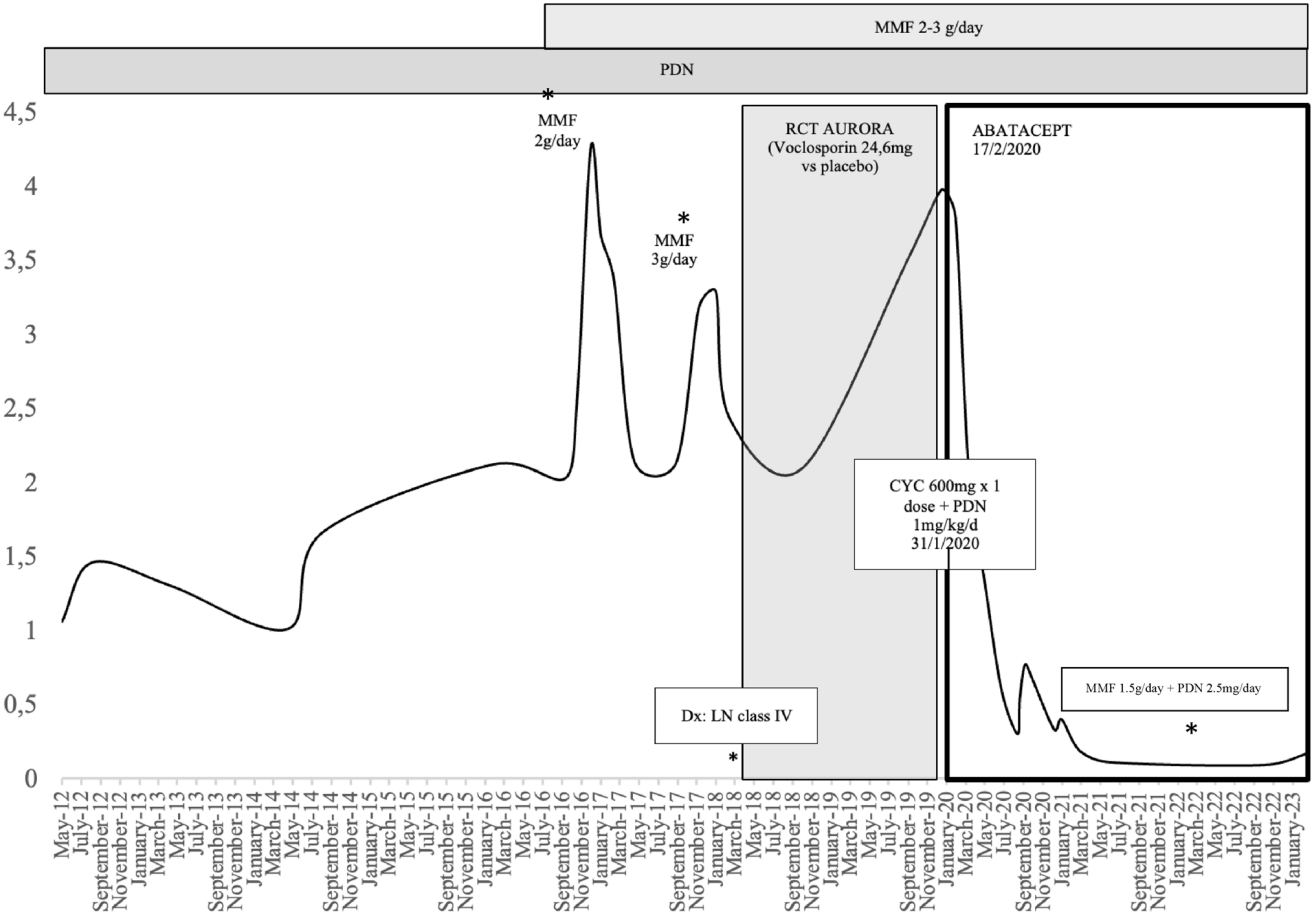
Proteinuria evolution of case 2 measured by UPCR

In this clinical context, in February 2020, it was decided to add off-label subcutaneous ABT (125 mg weekly), maintaining MMF 3 g/day and PDN 5 mg/day. After 6 months, CR was reached (UPCRs 0.3 g/g) with eGFR (CKD-EPI) 61 ml/min, that has been stable to date (February 2023), with normalization of complement levels and reduction of anti-dsDNA concentrations (23 UI/mL). ABT effect allowed to gradually reduce MMF (1.5 g/day) and PDN (2.5 mg) doses. As the only adverse event, the patient presented Herpes Zoster in the right lower limb. Proteinuria evolution from the beginning of follow-up in our center is illustrated in Fig. 2**.**

## Search strategy

We described the cases of two patients with refractory LN who experienced a full response after receiving Abatacept. We reviewed the literature regarding case reports and randomized controlled trials (RCT) written in English by searching medical journal databases in PubMed, Scopus, DOAJ and World of Science (WoS). A combination of terms “lupus nephritis”, “abatacept”, “systemic lupus erythematosus”, “refractory lupus nephritis” and “case report” was employed. We found one related case report of a patient with drug-intolerant LN and underlying monosomy 1p36 deletion syndrome who presented complete response after treatment with ABT [6]. We also found a retrospective analysis of 11 patients with refractory SLE, one of them with LN, who showed complete response with ABT [7]. According to second search, we identified 3 RCT that evaluated efficacy and safety of ABT in LN. They are further discussed in next section and summarized in Table 1.

**Table 1:** Summary of published literature on the use of abatacept in lupus nephritis

Case reports							
Study	Ethnicity	Biopsy class	Age, years	Total number	Treatment	Previous treatments	Results
Hosoda et al. [16]	Asiatic	Class IV	31	1	Abatacept 500 mg twice a month, and then 500 mg monthly Concomitant prednisone 30 mg tapered to 10 mg/day	Prednisone, MMF, hydroxychloroquine, tacrolimus, cyclosporine, rituximab, belimumab, mizoribine	Proteinuria decreased from 6.34 to 0.51 g/g at 12 months
Danion et al. [7]	–	Class IV	–	11 SLE patients (1 LN)	Abatacept (no dose specification) Concomitant prednisone 5 mg/day	CYC, rituximab and azathioprine	Proteinuria decreased from 2.64 to 0.4 g/g and haematuria disappeared at 6 months

Randomized clinical trials									
Study	Ethnicity	Biopsy class	Age, years (mean, SD)	Total number	Treatment	Standard of care	Patients	Follow-up	Outcomes
Furie et al. [26] (BMS-IM101075)	Mixed	III, IV, V	31 ± 9.5	199	Abatacept (2 doses)	MMF	99	52 weeks	CR^a^: 9.1%
					High dose (30/10)		99		CR^a^: 11%
					Low dose (10/10) Control		100		CR^a^: 8%
ACCESS, 2014	Mixed	III, IV, V	32 ± 10.1	134	Abatacept	CYC followed by azathioprine	66	24 weeks	CR^b^: 33%
					Control		68		CR^b^: 31%
ALLURE, 2018	Mixed	III, IV	33.1	505	Abatacept (30/10)	MMF and glucocorticoids	202	52 weeks	CR^c^: 35.1%
					Control		203		CR^c^: 33.5%

*SLE* systemic lupus erythemathosus, *LN* lupus nephritis, *MMF* mycophenolate mofetil, *CYC* cyclophosphamide, *CR* complete response

^a^Defined as follows: for renal funtion, if normal at screening, eGFR ≥ 90% of screening value and if abnormal at screening, ≥ 90% at 6-month, pre-flare value; UPCR < 0.26 g/g; inactive urinary sediment, assessed at day 337 and confirmed at day 365

^b^Defined as follows: for renal function, maintenance serum creatinine  ≤ 1.2 or ≤ 125% of its baseline; UPCR < 0.5 g/g

^c^Defined as follows: for renal function, maintenance of eGFR; UPCR < 0.5 g/g, absence of urinary cellular casts and prednisone ≤ 10 mg/day

## Discussion

The treatment of LN for more than 3 decades has been based on CYC and corticosteroids which have led to a significant improvement in the LN prognosis. However, their safety profile and the presence of refractory LN have motivated the search for new induction and maintenance schemes, either with the use of reduced doses of CYC (ELNT Trial) [8] or with other immunosuppressive drugs such as MMF (ALMS Trial) [9–11], calcineurin inhibitors (CNIs) [12–14] or a combination of both treatments [15]. Voclosporin, a new CNI, has been recently approved by the FDA as first-line therapy for LN in combination with standard therapy (MMF + PDN) since it was shown to be superior to standard of care in achieving renal CR [16, 17]. In patients with refractory SLE and LN, autologous and allogenic stem cell transplantation has led to a good control of active disease, but the high rate of relapses and the severe side effects observed make this approach unattractive to conduct further trials or routine use [18].

Various biologic therapies (RTX, BEL, tocilizumab (TCZ) or ABT) have been tested in patients with LN, as coadjutant to induction treatment with CFM + PDN or MMF + PDN, with very heterogeneous results.

The most relevant CT performed with RTX was the LUNAR, which failed to demonstrate greater efficacy compared to standard therapy in terms of renal response (UPCR ≤ 0.5, normal sediment and GFR within 15% of baseline values at 12 months). Despite this, due to the positive clinical experience and its low toxicity, RTX is commonly used off-label in patients with LN with good results [19, 20]. Regarding the use of TCZ in LN, only one small open RCT has been published with disappointing results [21]. BEL was the first biologic therapy (BT) approved for the treatment of LN thanks to the results of the BLISS-LN study (NCT01639993) [22], which primary endpoint was less strict than the one imposed in the LUNAR (UPCR ≤ 0.7 and stable eGFR [< 20% decrease compared to baseline value] or eGFR ≥ 60 mL/min at week 104). In the two cases presented, we tried to obtain, unsuccessfully, LN remission with all these BTs, so we opted to start treatment with ABT. The use of this molecule in animal models with SLE had showed benefits [23] and, based on these, it had been performed the first RCT with ABT in SLE patients. A post-hoc analysis of this study suggested its usefulness in LN [24].

For this reason, a RCT (ACCESS) was designed to assess its efficacy and safety in LN patients. Intravenous ABT was compared to placebo in patients with class III/IV LN, UPCR > 1 g/g and a positive ANA and/or dsDNA. In the active arm, ABT was added to the background induction therapy, which consisted of six doses of IV CYC 500 mg/2 weeks (based on ELNT trial regimen), followed by AZA and PDN at a dose of 60 mg/day for 2 weeks and then tapered to 10 mg/day over the next 10 weeks. The primary endpoint was defined as the proportion of patients who achieved CR at week 24. CR was defined as all the following criteria: UPCR < 0.5 g/g, serum Cr ≤ 1.2 or ≤ 125% of its baseline value with a minimum dose of PDN of 10 mg at week 12. Finally, there were no statistically significant differences between both groups in terms of PE at 6 months. CR was achieved in 33% patients from the active arm and in 31% patients from the placebo arm [25]. This trial explored only one dose regimen, based in the ABT dose approved for rheumatoid arthritis. It is possible that a higher dose might be more effective for LN. Regarding the background therapy, a combination with MMF could have been more effective. Also, it is possible that glucocorticoid therapy and multiple CYC doses have interfered with the mechanism of action of ABT. It should be noted that follow-up time was short, so that a longer observation period could have captured more patients achieving CR.

Simultaneously, another phase II/III RCT (BMS-IM101075) was published. The efficacy of intravenous ABT was tested in patients with class III/IV LN, complement C3 or C4 levels below the lower limit of normal or elevated anti-dsDNA titers, UPCR > 0.44 g/g and active sediment. Two different ABT doses were tested (low dose group: 10 mg/kg on weeks 0, 2 and 4, and thereafter one dose every 4 weeks; and high dose group: 30 mg/kg on weeks 0, 2, 4 and 8 and thereafter 10 mg/kg every 4 weeks) versus placebo. ABT was added to the background therapy with MMF (2–3 g/day) and PDN (30–60 mg/day for 4 weeks and then tapered to 10 mg/day over the subsequent 11 weeks, although this tapering was not mandatory). The PE at 12 months was the time to confirmed CR. CR was defined as the fulfillment of the following criteria in two consecutive visits: (1) < 10% reduction in eGFR compared to baseline, (2) UPCRs < 0.26 g/g, and (3) inactive urinary sediment. Despite a significant reduction in different markers of immune activity and proteinuria, there were no significant differences in reaching such a demanding PE, which was achieved by only 10% of the patients in the overall series [26]. However, in a subgroup analysis of patients with nephrotic-range proteinuria at baseline (*n* = 122), there was a 20–30% greater reduction in mean UPCR among those randomized to receive ABT versus placebo.

Wofsy et al., in a post-hoc analysis of this trial [27], criticized the primary endpoint and the lack of obligation for early PDN withdrawal. Regarding the entry criteria, BMS trial allowed enrollment of patients with a low UPCR (0.44 g/g), whereas other trials required UPCR > 1 g/g. Also, the requirement of a very low UPCR to define complete response eliminated some patients that would have met the more permissive target set for other relevant trials. The strict definition of stable renal function and the obligatoriness that the PE had to be achieved at two successive visits might have left out some patients who otherwise had met CR on day 365. After reanalyzing the BMS-IM101075 results, applying CR criteria used in the most important CTs up to that time (LUNAR, ALMS and ACCESS), Wofsy et al*.* concluded that ABT would have shown higher remission rates, especially in patients with nephrotic proteinuria.

Later, the ALLURE RCT (NCT01714817) analyzed the efficacy and safety of high-dose intravenous ABT (30 mg/kg monthly for 3 months, followed by 10 mg/kg monthly thereafter) versus placebo in patients with Class III/IV LN treated with MMF and PDN. CR was defined as eGFR stability, UPCR ≤ 0.5 g/g, and normal sediment with PDN ≤ 10 mg/day at 12 months. The study failed to achieve the primary endpoint, but patients treated with ABT achieved a faster and greater decline in anti-dsDNA titers and proteinuria, leading to a longer sustained CR in a post-hoc analysis [5].

It should be noted that all previous studies with ABT sought remission with induction treatment in patients with LN and were not specifically performed in patients who had already been shown to be refractory to standard treatment. This detail could explain its disappointing results, because the drug was added to a background therapy that has already been shown to be effective, so it is expected that its addition may not provide a relevant benefit in this group of patients.

The persistence of activity in the biopsies prior to the start of ABT in both patients reflects the lack of efficacy of all previous treatments and justifies the need to test new BT. Some of the data extracted from CT BMS-IM101075, in which ABT had shown better results in the subgroup of patients with nephrotic-range proteinuria, helped in decision making.

Regarding the combination with other drugs, and despite the lack of evidence, we opted to maintain TAC, MMF, and PDN in the first patient after the initiation of ABT and gradually reduce them until complete withdrawal months later. In the second patient, we didn´t add TAC because voclosporin had not shown any benefit and it didn´t prevent the patient from suffering a new renal flare. Reviewing the literature, we have not found any previous experience reported on the combined use of ABT with multitarget therapy in patients with SLE and/or LN. A small retrospective study of its use in patients with RA was published, with good results, although this study had not yet been published when we started treatment [28].

This study had some limitations, including its retrospective nature and the small number of cases reported. The extent to which previous biological therapies may have had a positive influence on disease control following the initiation of ABT is unknown.

## Conclusions

In our opinion, and based on our experience, we believe that ABT can be considered an effective and safe therapeutic alternative in the treatment of LN refractory to immunosuppressants and TB, especially in patients with proteinuria in the nephrotic range.

## Data Availability

The data underlying this article cannot be shared publicly due to the anonymization of the patient and for the privacy of individuals that participated in this work. Non-confidential data will be shared on reasonable request to the corresponding author.
